# Ultrasound-Guided Thoracic Paravertebral Nerve Block on Postoperative Pain, Quality of Life, and Recovery in Patients with Non-Small-Cell Lung Cancer

**DOI:** 10.1155/2021/6692815

**Published:** 2021-02-10

**Authors:** Cuijuan Zheng, Jiayu Wang, Shouxiang Xie

**Affiliations:** ^1^Department of Anesthesiology, The Affiliated Huaian No.1 People's Hospital of Nanjing Medical University, Huaian, Jiangsu, China; ^2^Department of Emergency, The Affiliated Huaian No.1 People's Hospital of Nanjing Medical University, Huaian, Jiangsu, China

## Abstract

**Objectives:**

Our study will investigate the effect of ultrasound-guided thoracic paravertebral block (UG-TPVB) on postoperative pain, quality of life, and enhanced recovery in patients with non-small-cell lung cancer (NSCLC) undergoing lobectomy surgery.

**Methods:**

Our study included 100 patients aged 52 to 75 years who underwent lobectomy surgery with pathological diagnosis of NSCLC. Patients received ultrasound-guided thoracic paravertebral block or general anesthesia with tracheal intubation. Patients' pain score was recorded on a numeric rating scale (NRS) 24 hours post operation. The total postoperative dosage of tramadol hydrochloride, length of hospitalization, quality of life (QoL), and inflammation levels were recorded.

**Results:**

Compared with patients who received general anesthesia with tracheal intubation, patients in the UG-TPVB group had lower postoperative NRS scores at 24 h (1.8 vs. 3.5, *P* = 0.035); the average 24 h postoperative NRS score of the UG-TPVB group is lower than that of the general anesthesia with tracheal intubation (4.6 vs. 5.3, *P* = 0.012), thus receiving less dosage of tramadol hydrochloride (221 ± 45 vs. 250 ± 38 mg, *P* < 0.01). Patients in the UG-TPVB group had better EORTC QLQ-C30 scores compared with patients in the general anesthesia with tracheal intubation group. The difference of length of hospitalization, hs-CRP, and IL-6 between two groups did not reach statistical difference (length of hospitalization 6.2 vs. 6.9 days, *P* = 0.055; hs-CRP: 7.1 ± 1.9 vs. 10.4 ± 6.6, *P* = 0.095; and IL-6: 71.3 ± 7.2 vs. 68.9 ± 8.7, *P* = 0.529). *Discussion*. NSCLC patients undergoing lobectomy surgery who received UG-TPVB had less postoperative pain, used less dosage of tramadol hydrochloride, and had better QoL.

## 1. Introduction

Lung cancer is among the leading cause of cancer death among both genders, taking up 25% of all cancer death according to the American Cancer Society's estimates for lung cancer in the United States for 2020 [1]. In general, around 13% of all lung cancers are small-cell lung cancer (SCLC), and around 84% are NSCLC. Surgery proves the best chance to recover from NSCLC. For most NSCLC patients undergoing surgery, the most commonly performed lung cancer surgery is lobectomy, which is the removal of the lobe of the lung that is affected by the tumor. Although general anesthesia with tracheal intubation is the most commonly used anesthetic method for NSCLC surgery, it has higher levels of inflammation and worse postoperative quality of life (QoL) compared with regional anesthesia [2]. Nerve block anesthesia such as thoracic paravertebral block (TPVB) has the advantages of reducing postoperative anesthetic consumption and reducing the inflammatory response and better postoperative survival rate for NSCLC patients [3]. TPVB has been widely used for decades, which is the technique of injecting local anesthetic adjacent to the thoracic vertebra near the spinal nerves emerging from the intervertebral foramina [4]. Traditional TPVB utilizes thoracic spine and disappearance of penetration as landmarks of the puncture site. Ultrasound-guided TPVB (UG-TPVB) has more advantages, such as pleural and lung tissue visualization and real-time visualization of puncture needle and catheter placement, which is safer to perform and provides excellent prolonged analgesic effect.

Surgery provides the best chance for NSCLC patients to recover; however, lobectomy is a complex operation. In this surgery, the entire lobe including the tumors is removed. Surgery can cause serious consequences, such as pain and lower QoL. In a study of operable, stage I, NSCLC patients undergoing lobectomy, nearly half of the patients had grade 3-4 treatment-related adverse events. 15% patients had grade 3 chest pain 15% and 7% had lung infections [5]. A clinical trial of 90 patients with resected NSCLC proved that UG-TPVB provided comparable levels of adequate analgesia for 24 hours after surgery and better analgesia after 12 hours. The UG-TPVB procedure could reduce the intraoperative fentanyl and postoperative morphine consumption [6]. The majority group of UG-TPVB did not need rescue morphine. Another study including 300 patients undergoing lung cancer resection using thoracotomy proved the opposite results. They concluded that using TPVB did not decrease the postoperative pain syndrome frequency [7]. Inconsistency about using UG-TPVB for NSCLC undergoing lobectomy still existed, and previous studies published no data about advantages of UG-TPVB on longitudinal postoperative pain in 24 hours, dosage of tramadol hydrochloride, inflammation, and QoL. Thus, our study is aimed at investigating the impact of UG-TPVB on postoperative pain using the numeric rating scale (NRS) score, dosage of tramadol hydrochloride 24 hours after surgery, postoperative inflammation levels using high-sensitivity C-reactive protein (hs-CRP), interleukin-6 (IL-6), and QoL using the European Organization for Research and Treatment of Cancer Quality of Life Questionnaire Core 30 (EORTC QLQ-C30).

## 2. Patients and Method

### 2.1. Study Design and Patients

In our study, we recruited patients aged between 52 and 75 years who were pathologically diagnosed of NSCLC at the Department of Anesthesiology, the Affiliated Huaian No.1 People's Hospital of Nanjing Medical University. Patients in the UG-TPVB group received 20 ml 0.25% adjunctive with anesthesia bupivacaine, and patients in the control group received general anesthesia with tracheal intubation (each group has 50 patients). Patients got no postoperative analgesic device. The patients' inclusion criterion was adult patients with pathological diagnosis of NSCLC undergoing lobectomy. Exclusion criteria were (1) patients diagnosed with another primary tumor within 2 years of recruitment, (2) patients with severe heart disease, uncontrolled cardiac arrhythmias, or hypertension, and (3) patients with cognitive impairment that make patients unlikely to be able to complete all study requirements. Patients' baseline clinical characteristics including age, gender, type of pathology, primary tumor, regional lymph nodes, body mass index, albumin, and hs-CRP were taken at recruitment. Patient postoperative pain, dosage of tramadol hydrochloride, levels of inflammation, and QoL were recorded.

### 2.2. Postoperative Pain Record and Management

Patients' postoperative pain scores were recorded using NRS. NRS is a pain scale that measures a patient's pain intensity. It is an 11-point scale for patient-recorded pain. It is based on patients' abilities to perform activities of daily living and can be used for adults and children more than 10 years old [8]. A rating 0 means no pain, 1-3 means mild pain, 4-6 means moderate pain, and 7-10 means severe pain. Patients were asked to give their NRS score 2 hours, 4 hours, 6 hours, 12 hours, and 24 hours postoperatively. When their score is ≥4, 50 mg tramadol hydrochloride was given intravenously for analgesia after patients' permission. We recorded the amount of tramadol hydrochloride in 24 hours after surgery.

### 2.3. QoL Assessment

Patients' QoL was assessed within 24 hours after surgery using the EORTC QLQ-C30. It is a patient-reported questionnaire developed for cancer clinical trials, clinical practice, and academic research. Patients need in average 10 minutes for finishing the EORTC QLQ-C30 questionnaire. The questionnaire has 30 questions in three scales, functional scale, symptom scale, and global health status. Most items use a four-item scale, namely, “not at all,” “a little,” “quite a bit,” and “very much.” Raw scores are transformed to a 0-100 scale. A high scale score represents a higher response level [9].

### 2.4. High-Sensitivity C-Reactive Protein and IL-6 Level

All of the blood samples were collected into the standard tubes in the same room at a temperature range of 22 to 24°C. Hs-CRP and IL-6 were measured by an immunoturbidimetric assay following standard laboratory procedures at the Department of Clinical Chemistry, the Affiliated Huaian No.1 People's Hospital of Nanjing Medical University.

### 2.5. Statistics

Descriptive statistical analyses of baseline characteristics were conducted for all continuous variables by mean values and standard deviations and categorical variables by counts (percentages). Comparisons of EORTC QLQ-C30 scores, NRS scores at 2 hours, 4 hours, 6 hours, 12 hours, and 24 hours after surgery, amount of tramadol hydrochloride dosage, hs-CRP, and IL-6 between UG-TPVB and general anesthesia with tracheal intubation subgroups were compared using Students' *t*-test. Longitudinal NRS scores were illustrated using a line chart. All analysis was performed using SPSS, version 16.0 (IBM Corporation, Armonk, NY, USA), and *P* < 0.05 was considered statistically significant.

## 3. Results

In total, 100 patients, all with unresectable NSCLC, consented to participate in the study from July 2017 to January 2019: 50 patients underwent UG-TPVB and 50 patients underwent general anesthesia with tracheal intubation. Baseline characteristics, such as gender, age, type of pathology, primary tumor (T stage), and regional lymph nodes (N stage), of the 100 patients are summarized in Table 1. Preoperative body mass index, albumin, and hs-CRP and IL-6 levels did not reach statistical difference between the two groups.

**Figure 1:**
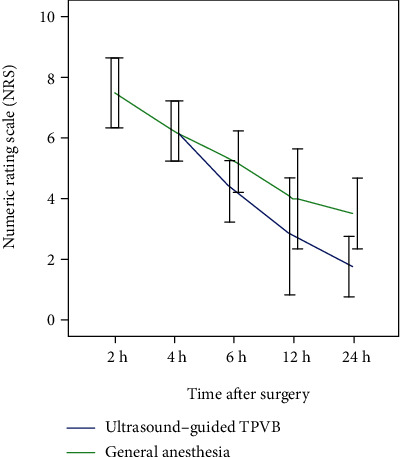
Patients' postoperative numeric rating scale (NRS) score in 24 hours. Patients in the UG-TPVB group has better NRS scores compared with the general anesthesia with tracheal intubation group. Although patients' NRS score at 2 hours, 4 hours, 6 hours, and 12 hours did not reach statistical difference, patients in the UG-TPVB group had lower postoperative NRS scores at 24 h (1.8 vs. 3.5, *P* = 0.035). The average 24 h postoperative NRS score of the UG-TPVB group is lower than that of the general anesthesia with tracheal intubation (4.6 vs. 5.3, *P* = 0.012).

**Figure 2:**
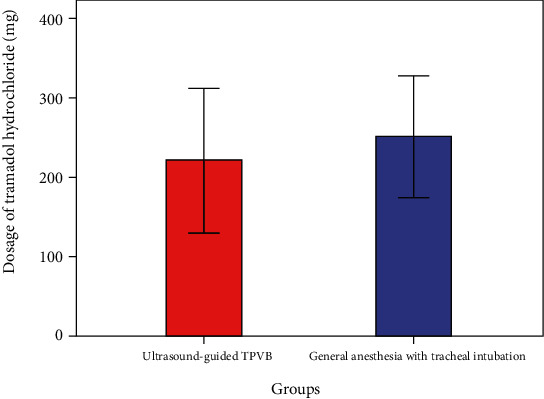
Postoperative consumption of tramadol hydrochloride in two groups. Patients in the UG-TPVB group received a lower dosage of tramadol hydrochloride compared with patients in the general anesthesia with tracheal intubation group (221 ± 45 vs. 250 ± 38 mg, *P* < 0.01).

The postoperative NRS scores at 2 hours, 4 hours, 6 hours, 12 hours, and 24 hours were illustrated using a line chart in Figure 1. Patients in the UG-TPVB group has better NRS scores compared with the general anesthesia with tracheal intubation group. Although the patients' NRS score at 2 hours, 4 hours, 6 hours, and 12 hours did not reach statistical difference, patients in the UG-TPVB group had lower postoperative NRS scores at 24 h (1.8 vs. 3.5, *P* = 0.035). The average 24 h postoperative NRS score of the UG-TPVB group is lower than that of the general anesthesia with tracheal intubation (4.6 vs. 5.3, *P* = 0.012). Patients in the UG-TPVB group received lower dosage of tramadol hydrochloride compared with patients in the general anesthesia with tracheal intubation group (221 ± 45 vs. 250 ± 38 mg, *P* < 0.01, Figure 2).

Table 2 shows the EORTC QLQ-C30 scores after surgery for NSCLC patients in two groups. Patients in the UG-TPVB group had a better emotional functional scale (40.3 ± 12.1 vs. 32.6 ± 10.4, *P* = 0.015), less pain (22.8 ± 5.8 vs. 49.9 ± 10.7, *P* < 0.01), and less insomnia (18.7 ± 16.2 vs. 31.0 ± 6.4, *P* = 0.023). The other functional scales, symptom scales, and global health status between the two groups did not reach statistical difference.

The difference of length of hospitalization, hs-CRP, and IL-6 between two groups did not reach statistical difference (length of hospitalization 6.2 vs. 6.9 days, *P* = 0.055; hs-CRP: 7.1 ± 1.9 vs. 10.4 ± 6.6, *P* = 0.095; and IL-6: 71.3 ± 7.2 vs. 68.9 ± 8.7, *P* = 0.529). However, patients in the UG-TPVB group had the tendency of shorter length of hospitalization and a lower level of hs-CRP level.

Thus, our data proved that in patients with NSCLC receiving UG-TPVB during lobectomy, the patient postoperative pain scores are decreased; patients had better QoL and had lower dosage of tramadol hydrochloride 24 hours after surgery. Patients receiving UG-TPVB tend to have shorter length of hospitalization and less inflammation compared with patients receiving general anesthesia with tracheal intubation.

## 4. Discussion

UG-TPVB is a safe analgesia procedure since ultrasound guidance is a recent technique that may offer several advantages. With the use of ultrasound (US), nerve block techniques are much safer, and a smaller dosage of local anesthesia is required [10]. Recent studies warrant the benefit of UG-TPVB [11]. TPVB is a technique which is easy to learn with a high success rate and a relatively low rate of serious complications like pneumothorax (0.5%) [12]. Previous study shows that paravertebral block has some benefits beyond providing analgesia [13]. Ultrasound-guided single injection TPVB gives effective postoperative analgesia in 24 hours after surgery [14]. In a study of 60 patients with the American Society of Anesthesiologists, status I and II undergoing elective laparoscopic cholecystectomy, the dose of intraoperative fentanyl, postoperative pethidine consumption, and the number of patients receiving pethidine were significantly lower in UG-TPVB groups compared to general anesthesia alone without any local anesthetic group (*P* < 0.05). Postoperative serum cortisol and blood glucose level showed a significant decrease in the UG-TPVB group compared with the general anesthesia group at 6 and 24 h postoperative (*P* < 0.05). The pain score by the visual analog scale and number of patients who experienced postoperative nausea and vomiting were significantly lower in the UG-TPVB group than patients in the general anesthesia group. Several studies proved that the postoperative pain score was significantly decreased in the TPVB group, and there was statistical significantly lower consumption of analgesic requirements compared with the general anesthesia group [15, 16]. Our study is in accordance with previous studies and provided further evidence that NSCLC patients undergoing lobectomy in the UG-TPVB group had less postoperative pain at 24 hours and received less tramadol hydrochloride dosage compared with patients receiving general anesthesia with tracheal intubation.

Our study is the first to evaluate the impact of UG-TPVB on postoperative QoL using the EORTC QLQ-C30 questionnaire. Our study confirmed that NSCLC patients undergoing lobectomy in the UG-TPVB group had a better emotional functional scale, less pain, and less insomnia compared with patients in the general anesthesia with tracheal intubation group. Sleep disorders had a high degree of coexistence with pain. Actually, the majority of patients with postoperative pain suffers sleeping impairment, including insomnia [17, 18]. Interestingly, some studies proved that insomnia may not only cause pain but also enhance chronic pain [17–19]. Thus, insomnia and postoperative pain promote and alter the processing of each other. Not surprisingly, there is overlap between pain and sleep disorder. NSCLC patients undergoing lobectomy in the UG-TPVB also had higher chances of a better emotional functional scale, which includes anxiety, depression, and impulsivity. Many patients experienced depression after surgery. Ongoing health problems, worry about future relapse, discomfort, and change in routine all can contribute to emotional health issues. Research shows that mental health issues can negatively affect patients' recovery after surgery [20]. Depression is a frequent cause of general morbidity in postoperative patients suffering from a wide range of complications. Also, depression after surgery can increase the perception or experience of postoperative pain [21]. Symptoms of emotional disorders include difficulty to make decisions, problems with memory, eating less or more than usual, sleeping disorders, loss of interest in activities, or feeling of despair or hopelessness with no specific reasons. Thus, contribution of UG-TPVB to pain, emotional scale, and insomnia may help recovery and needs to be further studied in larger clinical trials.

Patients in the UG-TPVB group had the tendency of shorter length of hospitalization and less inflammation. Severe inflammation is a condition that makes NSCLC patient recovery after surgery difficult. CRP is an acute phase protein that is regarded as one of the most commonly used clinical inflammatory marker used in routine practice [22]. Increased baseline or postoperative CRP levels could be used to test candidates of anti-inflammatory drugs. A hs-CRP test, which is more sensitive than a standard test, is a blood test that finds lower levels of CRP. A meta-analysis published in 2016 suggested that an increased baseline CRP level is associated with a statistically significant worse prognosis in lung cancer patients undergoing lobectomy [23]. A series of 1750 operable lung cancer patients who underwent complete resection proved that CRP levels at baseline and 3 days after surgery predict mortality [24]. IL-6 has been proved to be an inflammatory marker that predict poor survival for postoperative NSCLC patients [25]. We also tested the postoperative IL-6 level and found no statistical difference between two groups. Our results confirm the tendency of lower inflammation levels for NSCLC patients who underwent lobectomy in the UG-TPVB group. UG-TPVB has the tendency of reducing the length of hospital stay in NSCLC patients undergoing lobectomy.

Our study had several limitations. First, our study has a limited number of sample size, recruiting 100 patients. Second, patients were not blinded when they received UG-TPVB and needed to write informed consent. However, our study is the first to evaluate impact of UG-TPVB on longitudinal postoperative pain, QoL, inflammation, dosage of tramadol hydrochloride, and length of hospitalization in patients with NSCLC who underwent lobectomy. Further studies are needed in the field and trainings.

## 5. Conclusion

Use of UG-TPVB in NSCLC patients receiving lobectomy provided good postoperative analgesia and decreased the dose of tramadol hydrochloride used, with great safety and better QoL.

## Figures and Tables

**Table 1:** Baseline characteristics in the two groups.

Characteristics	Ultrasound-guided TPVB	General anesthesia with tracheal intubation
Number of patients	50	50
Age median (year) (range)	62 (53-75)	61 (52-72)
Sex: female	28 (56%)	25 (50%)
Sex: male	22 (44%)	25 (50%)
Type of pathology (%)		
Squamous cell carcinoma	20 (40%)	19 (38%)
Adenocarcinoma	28 (56%)	31 (62%)
Adenosquamous carcinoma	2 (4%)	0
Primary tumor (T), *n* (%)		
T1	28 (56%)	25 (50%)
T2	19 (38%)	20 (40%)
T3	3 (6%)	4 (8%)
T4	0	1 (2%)
Regional lymph nodes (N), *n* (%)		
N0	26 (52%)	28 (56%)
N1	16 (32%)	18 (36%)
N2	8 (16%)	4 (8%)
Body mass index, mean ± SD (kg/m^2^)	26.9 ± 0.9	25.8 ± 1.0
Albumin, mean ± SD (g/l)	29 ± 4.2	29 ± 3.7
High-sensitivity C-reactive protein, mean ± SD (mg/l)	4 ± 1.2	3.2 ± 1.5

**Table 2:** The European Organization for Research and Treatment of Cancer Quality of Life Questionnaire Core 30 (EORTC QLQ-C30) in two groups.

	Ultrasound-guided TPVB (*n* = 46)	General anesthesia with tracheal intubation (*n* = 45)	*P* value
Functional scale			
Physical	68.1 (18.4)	69.1 (20.5)	0.883
Role	62.3 (18.7)	69.6 (21.0)	0.299
Emotional	40.3 (12.1)	32.6 (10.4)	0.015
Cognitive	63.8 (19.3)	61.0 (26.0)	0.684
Social	50.0 (17.2)	50.9 (21.9)	0.858
Symptom scale			
Fatigue	39.5 (11.2)	34.1 (6.2)	0.149
Nausea/vomiting	8.1 (8.0)	10.8 (12.1)	0.446
Pain	22.8 (5.8)	49.9 (10.7)	<0.01
Dyspnea	42.4 (18.6)	36.9 (18.8)	0.295
Insomnia	18.7 (16.2)	31.0 (6.4)	0.023
Appetite loss	31.8 (12.3)	29.7 (6.6)	0.457
Constipation	37.0 (28.5)	38.1 (15.6)	0.894
Diarrhea	10.6 (4.8)	13.3 (5.1)	0.253
Financial difficulty	22.2 (13.9)	28.8 (8.1)	0.108
Global health status	52.6 (11.6)	50.3 (13.9)	0.654

Data are presented as the mean (standard deviation). Higher scores indicate better functioning in those functional scale and global health status and worse symptoms in the symptom scale.

## Data Availability

Data are available from the corresponding author on reasonable request.
